# “Just give it to us straight!”: a qualitative analysis of midwestern rural lung cancer survivors and caregivers about survivorship care experiences

**DOI:** 10.1007/s11764-023-01445-7

**Published:** 2023-08-26

**Authors:** Marquita Lewis-Thames, Samuel Keimweiss, Anna Gurolnick, Shakira J. Grant, Jessica Burris, Jamie Studts

**Affiliations:** Northwestern University; Northwestern University; Northwestern University; University of North Carolina at Chapel Hill; University of Kentucky; University of Colorado Anschutz Medical Campus

**Keywords:** Cancer survivors, rural health services, qualitative research, lung cancer, caregivers

## Abstract

**PURPOSE:**

We assessed the experiences of rural lung cancer survivors and caregivers to understand and identify barriers to posttreatment survivorship care management.

**METHODS:**

From May 2021-June 2022, we conducted semi-structured interviews with a purposively sampled cohort. Participants were either posttreatment lung cancer survivors (within 5 years of their last active treatment) or caregivers of a lung cancer survivor. Interviews probed participants regarding survivorship care knowledge, implementation, and navigation. Two analysts inductively coded verbatim transcripts and conducted a thematic analysis.

**RESULTS:**

We interviewed N = 21 participants: lung cancer survivors (76%) and caregivers (24%). Participants self-identified as Non-Hispanic White (100%), were at least 65 years old (77%), identified as male (62%), and previously smoked ≥ 5 packs of cigarettes/day (71%). The perspectives of survivors and caregivers were similar; thus, we analyzed them together. Themes related to survivorship care included: (1) frustrations and uncertainty regarding unexpected barriers, (2) strategies to improve the delivery of posttreatment information, (3) strategies to remain positive and respond to emotional concerns of survivorship care, and (4) the impact of engaging and patient-centered care teams.

**CONCLUSION:**

Given the limited access to lung cancer care resources in rural communities, our findings reveal that following a survivorship care program or plan requires a high level of individual resilience and community/interpersonal networking.

## INTRODUCTION

Lung cancer is the most commonly diagnosed cancer among men and women. Estimates suggest that nearly 660,000 Americans are living with lung cancer.[1] Despite the increasing availability of novel life-prolonging therapies, the 5-year survival rate for lung cancer remains one of lowest for all cancers, making it cancer with high incidence *and* mortality.[2] Additionally, more than half (55%) of all adults living with lung cancer in the United States have been diagnosed in the past 5 years, demonstrating the low survival rate of lung cancer patients.[3] The burden of lung cancer is particularly heavy for rural cancer survivors and their caregivers. For example, rural adults with lung cancer have a median survival that is 12 months less than urban lung cancer survivors.[4] Trends toward improving 5-year survival rates for adults with lung cancer in rural communities have trailed behind those of their urban counterparts for almost 40 years.[2] Given the rural lung cancer burden, rural survivors and their caregivers are particularly vulnerable, and if left unaddressed, gaps in rural and urban survival rates will likely widen.

Causes for rural-urban disparities in lung cancer survival comprise individual- and community-level risk factors. Individual-level risk factors include a higher prevalence of smoking among rural residents compared to urban residents.[5-8] Among community-level risk factors, rural residents are more likely than urban residents to have reduced access to quality healthcare resources, especially with the nationwide closure of over 160 rural hospitals in the past 15 years.[9, 10] Compared to urban areas, rural areas have fewer low-dose computed tomography scans for lung cancer screening, lower screening rates, and less access to specialists like oncologists, all of which contribute to diagnoses of more advanced diseases with a lower chance of survival.[11-13] Increasing rurality is also associated with delays in diagnosis, which can lead to more advanced and symptomatic disease at initial presentation; furthermore, rural residents are more likely than their urban counterparts to experience treatment delays.[4, 14, 15] These delays in diagnosis and treatment initiation are associated with poor survivorship outcomes, including lower quality of life and functional well-being.[16, 17] In addition, rural residents are further disadvantaged due to the interplay of sociodemographic factors such as individual experiences with persistent poverty, neighborhood-level deprivation, and high financial burdens.[18-20] Considering the individual and community level barriers to quality rural cancer care, and that survivorship care is particularly challenging for rural residents, strategies that focus on medical and non-medical (e.g., structural inequities, social drivers of health) solutions are critical to eliminating geographic disparities.

The 2006 Institute of Medicine (IOM) report, *From Cancer Patient to Cancer Survivor: Lost in Transition,* played a critical role in the nationwide conversation about long-term cancer survivorship, providing 10 recommendations to improve follow-up cancer care.[21] While the importance of certain recommendations from the 2006 IOM report continues to be evaluated and debated (e.g., survivorship care plans), enthusiasm for key elements of a survivorship care plan remains (e.g., treatment symptom report). In 2019, the Commission on Cancer recommended a focus on comprehensive survivorship care programs including, but not limited to, treatment summaries, screening for new cancers, psychological support and psychiatric services, and financial support services.[22] An additional emphasis was placed on engaging an expansive multidisciplinary care team composed of but not limited to, physicians, nurses, social workers, nutritionists, and other allied health professionals. In addition to clinical services related to monitoring and surveillance, other supportive or palliative care services that commonly compose the survivorship care program for adults with lung cancer include pain management, smoking cessation, and emotional support.

Related to implementing an interprofessional, multi-serviced survivorship care program recommended by the Commission on Cancer, rural lung cancer survivors and their caregivers are likely disadvantaged. Recommended services of a comprehensive survivorship care program are likely distal and located in an urban center.[23] Considering the comprehensive representation of services provided in a survivorship care program, adherence is likely threatened by transportation and financial barriers and is complicated by a reduced understanding of the perceived importance of the services.[24] Residing in rural communities is associated with a 78% increase in missed follow-up care visits compared to urban residents. [16, 23] Notably, COVID-19 impacted access to important survivorship program services. For rural patients with cancer, cancer screenings, genetic counseling, tobacco cessation counseling, and pharmacotherapy decreased compared to pre-pandemic volumes.[14, 23, 25] Concurrently, many rural hospitals and clinics reported temporarily closing due to COVID-19-diagnosed clinicians and staff, low patient volume, and associated financial difficulties.[25] To summarize, there are notable barriers to rural lung cancer survivors’ access to and engagement with survivorship care programs, as the Commission on Cancer recommended. Without practice training and policy reform to improve service access, these barriers will continue to exacerbate existing rural-urban lung cancer disparities.

As lung cancer survivorship care is not "one size fits all," understanding how survivorship care is delivered to and received by rural lung cancer survivors may provide important insights for improving survivorship outcomes for rural lung cancer survivors. However, there are no assessments of posttreatment survivorship delivery from the perspectives of rural lung cancer survivors and caregivers of lung cancer survivors. To fill this gap in the literature, we examined survivorship care experiences and implementation among rural lung cancer survivors and caregivers receiving care from rural and rural-serving Midwestern hospitals. This qualitative investigation may provide insights into strengths and opportunities for delivering survivorship care for rural lung cancer survivors. Further, findings can inform best practices for implementing survivorship care programs with rural lung cancer survivors and lend to improving survivorship outcomes and eliminating lung cancer survivorship disparities.

## METHODS

### Study design

The institutional review board at Northwestern University (Study STU00213828) approved the study protocol for qualitatively assessing rural community member perspectives of personal and interpersonal experiences in lung cancer survivorship care management and delivery. This study followed the Standards for Reporting Qualitative Research (SRQR) guidelines for qualitative studies.[26, 27] This study applied grounded theory to identify themes and subthemes from participant narratives.

### Study population and recruitment

Rural lung cancer survivors were eligible if they: (1) self-identified as rural, (2) finished active treatment within the last five years, (3) were at least 55 years old, (4) were English-speaking, and (5) were not institutionalized. Caregivers of rural lung cancer survivors were eligible if they: (1) provided care for a lung cancer survivor that self-identified as rural, (2) provided care for a rural lung cancer survivor that finished active treatment within the last five years, (3) were at least 18 years old, (4) English-speaking, and (5) not institutionalized. The participation of a caregiver was not dependent on the enrollment of a survivor. In sum, we screened 41 potential participants, of which 21 participants were interviewed. Reasons for survivors declining participation included prioritizing health care concerns (e.g., restarting treatment) or lung cancer progression. No eligible and available caregiver declined participation.

We used purposive sampling to recruit rural lung cancer survivors and caregivers of rural lung cancer survivors. Most participants were initially identified and approached by a nurse or hospital administrator from academic-affiliated cancer centers or a rural-serving community hospital. In an effort to increase racial and ethnic diverse populations, a small subsample of participants was approached through community-based leaders that serve rural communities or snowball sampling methods–wherein an enrolled participant refers to other potential participants.^16^ We also employed passive recruiting strategies (e.g., flyers and handbills). There were no pre-existing relationships between members of the study team and participants.

### Study setting

We conducted in-person or telephone interviews at the request of the participant. We conducted all in-person interviews at a convenient location suggested by the participant (e.g., the participant's home). Participants were encouraged to complete interviews in a private location. The research team also conducted all interviews in a quiet private location.

### Data collection

We developed a semi-structured interview guide (Supplemental Online resources). Interview guides for caregivers and survivors were similar but adapted to the participant's role. The interview guide included a brief description of posttreatment survivorship care, follow-up care visits, the interview process, and the study objectives, and emphasized the importance of engaging community members in the research process. The interview guide questions included topics and prompts related to survivorship care, such as management, knowledge, experiences, and information gathering.

Interviews were conducted by the Primary Investigator (PI), a qualitative and rural health researcher with over 10 years of experience in qualitative interviewing and coding (MWLT), and a trained graduate student research assistant who has two years of training and work experience with qualitative research (AG). During the interview, the interviewers shared limited personal details (e.g., name, personal interest in rural health) to minimize bias and influence.

The PI and research assistant screened all participants for eligibility before their interview. Eligible participants were assigned a unique identifier and scheduled for an in-person interview at a location convenient for the participant or over the telephone. All participants provided verbal consent to a single interview immediately before the interview. After consenting, the interviewer collected demographic data from each participant. All interviews followed the interview guide and the interviewer dictated field notes during and after the interview when appropriate. The average length of an interview was 38 minutes. With the permission of the participant, all interviews were audio recorded. Recordings were transcribed verbatim by a third-party professional transcriptionist. AG assessed the accuracy of all transcripts by comparing sections of the transcript with the audio recording. Transcripts and audio recordings that were excessively discordant were re-transcribed by the transcription service provider. AG also ensured that all transcripts were de-identified.

### Data analysis

We used NVivo 12 (QSR International, Melbourne, Australia) for project management and to facilitate data analysis.[28] Following a thematic analysis approach, transcripts were double-coded inductively–developing codes as the transcripts were read–to identify salient content and relevant themes.[29] Each transcript was double-coded to improve the reliability of the findings. Each transcript was individually coded by a paired team of research team members (AG and SK). Paired coders conducted consensus meetings regularly to compare and discuss emergent codes, develop the codebook, and monitor saturation. Thematic saturation is an iterative review process in which text is coded until no new codes emerge.[30, 31] The PI joined the paired coders to mediate any coding disagreements. Themes and subthemes were developed by identifying code frequency and similarity using cluster maps and hierarchy charts. The trustworthiness of the study was established via multiple methods, including an audit trail (e.g., record keeping of raw data, field notes, transcripts), peer debriefing, and team consensus on themes.[32, 33]

## RESULTS

We interviewed 16 lung cancer survivors and 5 lung cancer caregivers (Table 1). All survivors and caregivers were Non-Hispanic White race. Most survivors and caregivers were 65 years old or older, married, male gender, received at least a high school diploma, not currently smoking, had an annual income of $49,999 or less, and traveled less than 30 minutes to their primary care provider or the oncologist, and consulted a nurse or oncologist for survivorship care information. Compared to caregivers, survivors were older, had lower levels of education, lifetime smoking history, had a lower annual income, and consulted members outside of a clinical care team (e.g., support group, Facebook) for survivorship care information.

Survivors and caregivers reported four major themes regarding their experiences with survivorship care: (1) frustrations and uncertainty regarding the unexpected barriers, (2) strategies to improve the delivery of posttreatment information, (3) strategies to remain positive and respond to emotional concerns of survivorship care, and (4) the impact of engaged and patient-centered caregivers and care teams. Tables 2-5 provide exemplar quotes that represent each theme and corresponding subthemes.

### I. Frustrations and uncertainty regarding unexpected barriers

A commonly discussed survivorship care concern was the frustrations and uncertainty regarding unexpected barriers (Table 2). This theme emphasizes that while participants accepted some barriers–*unexpected barriers* punctuated a frustrating and uncertain survivorship care experience. Namely, survivors and caregivers described those unexpected barriers arising from 1) adhering to posttreatment survivorship care recommendations and 2) challenges completing daily tasks that lead to frustration and uncertainty.

#### Barriers to adhering to clinical posttreatment survivorship care recommendations.

Among the identified barriers limiting adherence to survivorship care recommendations included limited survivorship care information, poor preparation for daily posttreatment care, trouble obtaining medications, and poor care coordination. As a result of the frustrations and uncertainty that arose due to barriers to adhering to posttreatment survivorship care recommendations, survivors likely delayed their care and experienced cancer-related distress or general anxiety. A survivor described a barrier to adhering to care recommendations as, "I [don't] blame it on the doctor… but I think they could have put a little effort or give me a list [of providers to contact]."

Similarly, caregivers mentioned barriers to helping survivors adhere to their survivorship care. One caregiver commented on their frustrations with receiving timely follow-up care appointments for a cancer survivor: "[The provider] was two hours late…But we knew we were right because we - we - both - both of us had it written down on our calendars."

#### Barriers to daily living.

In addition to barriers impeding adherence to posttreatment survivorship care recommendations, survivors and caregivers commonly commented on uncertainty regarding unexpected barriers to carrying out activities of daily life. Frustrations and uncertainty stemming from barriers to daily living often included challenges associated with treatment-related side effects and getting acclimated to day-to-day living as a posttreatment survivor. A survivor succinctly describes their functional limitations as,

"You don't really realize that you're gonna need that help with your food, and eating, and your bedsheets, and getting in and out of the bath or whatnot, or going on - doing your errands, picking up your medicine."

Notably, caregivers reported barriers to the daily activities of the survivor, not their own daily activities. For example, a caregiver said, "I made many meals for her. Um, because, you know, when they're done with their treatments, and they go home, they don't feel well. They're not going to cook for themselves."

### II. Strategies to improve delivery of posttreatment information

Many participants also discussed how they preferred to receive their posttreatment survivorship care information. In this context, posttreatment survivorship care information included but was not limited to provider care recommendations, personal health information, and supportive care service recommendations. Participants preferred to receive this information 1) as clear and specific care recommendations and 2) in a way that was convenient, direct, and delivered through multiple modalities (Table 3).

#### Communication needs to be clear and specific.

Specific and clearly communicated recommendations were often described as easy to follow and responsive to the survivor’s needs. For example, a survivor reflected on their conversation with a provider regarding their survivorship care as, "[My provider is] very good about sitting there and explaining everything and what will be done, how things will be done." Furthermore, participants discussed desired care information about personal needs and care expectations. Regarding care expectations, a survivor "wished" that they had been told that, "if you're going to have difficulty breathing, then you will be given an inhaler, or you can have respiratory therapy."

#### Modes of communication were diverse but needed to be convenient and direct.

Participants appreciated receiving their posttreatment information through varied modes. Primarily, they preferred to receive this information in-person with supplemental written information that included patient-specific care recommendations (e.g., specific care plans) and more general health information. Participants were receptive to receiving posttreatment information via telephone for patient-specific needs (e.g., medical information) or more generalized tasks (e.g., scheduling). For example, a caregiver explained their satisfaction with communicating with a nurse coordinator via telephone and digital photographs, "I would get a hold of [the nurse coordinator] …and tell her about the wound. And…what was nice, I snapped some pictures of it, and I sent it to her. …She said, yeah, that's - that's the way it should be." Another survivor appreciated receiving information "in the doctor's office" and through "follow-up calls."

### III. Strategies to remain positive and respond to emotional concerns of survivorship care

Anxiety and stress related to cancer survivorship were common concerns; however, survivors and caregivers worked to remain positive about their conditions and cancer management (Table 4). Multiple strategies were used to manage a positive disposition and related emotional concerns, yet participants identified that a) self-reliance, b) a responsive clinical care team, and c) strong social networks were helpful.

#### Self-reliance.

The subtheme, self-reliance, highlights a sense of independence, determination, and grit rooted in maintaining positivity and adherence to care plans despite the challenges associated with survivorship care. Namely, survivors preferred self-reliance over seeking support from social networks or support groups. A survivor commented that, "My family…I just don't want to burden anybody. I'm too strong for it. I don't sound very strong right now, but I really am." Survivors also felt the need to be self-reliant as it related to their attempt to stay positive and handle their emotional concerns in the face of pain or anxiety. As one survivor commented,

"I could feel myself getting kind of depressed, and I can't allow me to stay in that state of mind…when you wake up each morning, you have two choices; you can have a good day, or you can have a bad day."

Similarly, caregivers acknowledged their need to be self-reliant and to encourage independence among their care recipients especially as it relates to encouraging the survivor's cancer care management. A caregiver recalled that "…sometimes support groups are not success stories" and that survivors would have to "find [help] where [they] can."

#### A responsive care team.

Regarding the ability to remain positive or handle emotional concerns, a responsive care team provided information, specific guidance, instructions, and a source of authority and companionship. The care team most often included nurses and oncologists but could also include radiologists, pulmonologists, and surgeons. Caregivers and survivors identified care teams that helped them with their stress, "made [them feel] at ease," gave them "hope," and inspired survivors with reaffirming comments. A survivor mentioned, "My whole team…the way they was treating me, it make me… feel that they they work with me." Another survivor recalled that, "[My provider] wasn't telling me to take these tests just because to make a bunch of money…You know, he was telling me to take these tests because he cared." As one survivor stated, "anyone who walked in, anybody you talked to, anybody that you had [a] question with, they was right there to answer it." Importantly, a responsive care team helped caregivers and survivors remain positive and "comfortable." A caregiver reported that their responsive care team tole them that, “'Yeah, we can kick [cancer’s] butt. We can do this."

#### Strong social networks.

Caregivers and survivors identified that strong social networks with friends, family, and neighbors were critical for their posttreatment survivorship care management and emotional concerns. This was particularly important during the early years of the Covid-19 pandemic, as commented, "What really hurt me was this COVID because it put me in isolation." A survivor replied "the support that you're getting, it's more important. To me, it was…the best medicine." A caregiver described that having a strong "[family] support system is very important." Similarly, a survivor commented on support from their sister as,

"If I'm eating correctly, and if I'm not my one sis brings me cabbage rolls. I know I - she's know I like them. But, uh, they - they just pretty much check in to see if I'm sleeping. They want to make sure because I have four stairs that goes down from the upper - you know, my kitchen, down to my living room, and, uh, they're concerned, me walking up and down the stairs."

### IV. The impact of engaging and patient-centered care teams

Engaging and patient-centered formal (i.e., clinical) and informal (i.e., neighbors, family) care teams were critical for posttreatment survivorship care management. Participants often identified the benefits of engaging and patient-centered care teams for managing: a) pain and b) stress (Table 5).

#### Pain management.

Caregivers and survivors stressed the importance of an engaging and patient-centered care team as responsive to managing pain related to treatments and therapies throughout posttreatment survivorship care. As an example of an unresponsive care team, a survivor mentioned that, "I kept telling [my provider] I was in so much pain, and he didn't want to give me any more pain medications. That's why I wound up in the emergency room". The same survivor provided an example of an informal care team member engaging in pain management in her comment, "If it wasn't for my daughter-in-law, I would probably starve to death because I was in a lot of pain." Similarly, a caregiver described their posttreatment care as largely consumed with pain management, commenting, "And it really hurts me to see her suffering like that…And just now, we're dealing with pain."

#### Stress management.

Stress management refers to, but is not limited to, managing stress related to receiving test results, recurrence, posttreatment care management, adjusting to life after cancer, and caregiver strain.Caregivers and survivors that valued stress management support from their engaging and patient-centered care team described them as feeling "secure instead of having to worry," without "doubt in their…words," and "somebody was looking out." A survivor commented on a member of their formal care team, "…he would tell me that even - not to worry, even if it did come back, there's other things that can be done. And I mean, just made me feel more secure instead of having to worry about it all the time.”

## DISCUSSION

We interviewed lung cancer survivors and caregivers of patients with lung cancer from rural areas in a Midwestern state and identified four themes related to their posttreatment survivorship care. Commonly, rural lung cancer survivors and caregivers were concerned about (1) frustrations and uncertainty regarding unexpected barriers, strategies to improve the delivery of posttreatment information, (3) strategies to remain positive and respond to emotional concerns of survivorship care, and (4) the impact of engaging and patient-centered care teams. Given persistent rural-urban disparities in posttreatment survivorship outcomes, this study's findings advance our understanding of the needs and concerns of posttreatment lung cancer survivors and caregivers of posttreatment lung cancer survivors.

A common thread weaved through several of our themes focused on emotional concerns related to survivorship care management. In our study, a responsive formal (e.g., providers, caregivers) and informal (e.g., friends, neighbors) care team were responsive to a survivor’s emotional concerns. Emotional concerns were often related to uncertainty arising from unexpected barriers and stress management and inspired self-reliance in survivors and caregivers. Notably, due to the higher cancer mortality and lower survival, lung cancer is associated with an increased rate of distress.[34] Yet, high-quality patient-provider communication is associated with significantly reduced odds of distress (OR = 0.15, 95% CI = 0.02–0.92). [35] Cancer-related distress is psychological distress or anxiety arising from a diagnosis and disease management. As more than 80% of all rural counties are considered mental health shortage areas,[10] rural residents have major access barriers to evaluating and treating cancer-related distress. Multiple studies punctuate the intersectionality of rural cancer and mental health burden as a public health crisis, yet, interventions and strategies to improve the timely assessment and management of cancer-related distress for rural older cancer survivors remain under-researched.[36-38] Given the high proportion of lifetime smokers in our sample, the absence of communication regarding smoking cessation programs was noted. Smoking cessation is critical to survivorship care management. This is possibly explained by previous findings reporting an apprehension to discuss smoking cessation in rural communities due to smoking stigma.[39, 40]

Another common thread identified in our themes focused on the communication and the delivery of posttreatment survivorship care information. Collectively, participants preferred to receive clear, patient-centered, and exhaustive posttreatment survivorship care information that emphasized stress and pain management resources, provided encouragement, and was relayed through different communication channels. There is increasing research and practice-level attention on survivorship care communication, partly due to the mixed findings on the utility of survivorship care plans and their association with improved survivorship outcomes.[24, 41, 42] Lewis-Thames et al.,[43] investigated patient-provider survivorship care communication with rural active and posttreatment survivors. Therein, less than half of the survivors received "in detail" communication about late or long-term side effects of cancer treatment (32%) and emotional or social needs related to cancer and lifestyle or health recommendations such as diet (47%). Relatedly, in an investigation of rural primary care providers’ understanding of survivorship care plans, 48% were unsure of their role in facilitating the care plan.[44] Collectively, these findings complement the current study and reveal barriers to exhaustive posttreatment survivorship communication for rural residents.

We observed in our themes that survivors relied on an integrated care team with both clinical and informal care teams. Consistent with our findings, rural residents with complex care, like survivorship care, report an increased reliance on informal caregivers to overcome reduced access to qualified health resources.[45] In an assessment of rural cancer survivors, a spousal informal caregiver increased mental health quality of life.[46] There is a paradigm shift in the literature–largely in Alzheimer’s and Dementia research–from the focus on a single caregiver to caregiving networks that support patients with complex care. Caregiving networks typically include multiple supporters of a patient and are often informal, contain a primary generalist or caregiving core group, are geographically dispersed, larger for patients with chronic conditions, and recognize that patients can also overlap in caregiving roles.[47-50] For rural cancer survivors that must rely on caregiving networks, it is possible that advice from larger networks may conflict with the recommendations of a less engaging clinical care team. But additional research is needed to explore this hypothesis.

While we interviewed rural lung cancer survivors and caregivers, our findings have clinical, research, and policy implications for improving posttreatment survivorship care management for rural cancer survivors across cancer types. This study and others have found that rural cancer survivors often minimize their distress.[51, 52] Therefore, continuing education for providers and supportive oncology staff discussing cancer-related distress with rural survivors should include strategies to normalize emotional concerns and reduce stigmas associated with receiving mental health support. Furthermore, current approaches to improving access to mental health and related survivorship care (e.g., telehealth-enhanced survivorship care) resources are often developed in an urban context.[10, 24] Findings from this study support developing interventions focused on cancer-related distress that are developed or adapted through research-community partnerships that engage rural community members through the research development and implementation process.

## LIMITATIONS

While the sample population was diverse in some respects (gender, income, education), it was homogeneous in other respects (race/ethnicity). Our racially homogenous sample reflected the racial and ethnic makeup of the counties represented in our sample, as the counties’ Non-Hispanic White population was 74%-90% of the general population. Also, our racially homogenous sample reflected limited opportunities to engage more diverse audiences through more community-based recruitment due to COVID-19 precautions. To this end, caution should be taken in generalizing these findings, to racially and ethnically diverse rural regions. Future studies should oversample racial/ethnic diverse residents to improve generalizability. Participants self-identified as rural, and some participation bias likely threatens our findings. The accuracy of rural and urban classification in health outcomes research continues to be a point of inquiry, and findings from this study add to the ongoing research to identify the most accurate representation of rural and urban areas in cancer care research. Future research should confirm our findings with alternative, more objective measurements of rurality. Although interviews were facilitated by trained Research Assistants and an experienced qualitative researcher using rigorous research protocols and procedures, data interpretation may contain participant and researcher bias. Similarly, codes used to develop themes and, ultimately, the study findings are limited by the researchers' judgment and restricted to concepts and interpretations of meaning deemed appropriate by the consensus of the interviewing researchers (AG and ML-T).

## CONCLUSION

For rural posttreatment lung cancer survivors and caregivers from rural Midwestern counties, participants emphasized frustration and uncertainty in light of unanticipated barriers related to daily tasks and their care. Additionally, participants commonly endorsed clear and specific posttreatment care information delivered through diverse media (e.g., written and telephone calls with the provider) as important strategies to improve their posttreatment care information delivery. Being self-reliant, having a responsive care team, having and access to strong social networks helped participants remain positive in the face of their care and in order to address ongoing emotional concerns. Participants preferred a patient-centered and engaged care team that gave special consideration to the patient's pain and stress management needs. Insights from this study shed light on potential strategies to improve survivorship care management for rural residents-a population particularly burdened by high lung cancer incidence and mortality. Future research on rural lung cancer disparities and intervention development should be built on and with the voices of rural cancer survivors and caregivers.

## Figures and Tables

**Table 1:** Population characteristics

	Overall	Survivor		Caregiver		
	N=21	n=16	%	n=5	%	p-value
**Sociodemographic characteristic**						
**Age**						
45-54	2	0	0%	2	40%	
55-64	2	2	13%	0	0%	
65-74	9	8	50%	1	20%	
75 and over	8	6	38%	2	40%	
**Race**						
Non-Hispanic White	21	16	100%	5	100%	
**Marital Status**						.47
Married	11	7	44%	4	80%	
Never Married	1	1	6%	0	0%	
Widowed	4	4	25%	0	0%	
Unreported/Declined	2	1	6%	1	20%	
Divorced	3	3	19%	0	0%	
**Gender**						.04
Male	13	10	63%	3	60%	
Female	8	6	38%	2	40%	
**Education**						.05
Some HS	5	5	31%	0	0%	
HS Diploma	6	5	31%	1	20%	
Associate/GED	2	2	13%	0	0%	
Bachelors	3	1	6%	2	40%	
> Bachelors	4	3	19%	1	20%	
Unreported/other	1	0	0%	1	20%	
**Smoking (life)**						.77
≥5 Packs	15	13	81%	2	40%	
<5 Packs	5	2	13%	3	60%	
Unreported	1	1	6%	0	0%	
**Smoking(now)**						.33
Yes	2	1	6%	1	20%	
No	18	14	88%	4	80%	
Unreported	1	1	6%	0	0%	
**Insurance**						
Private	13	9	56%	4	80%	
Medicare/caid	18	15	94%	3	60%	
**Income**						.12
<$50,000	7	6	38%	1	20%	
$50,000-$99,999	6	5	31%	1	20%	
$100,000 or more	3	2	13%	1	20%	
Not Sure	1	0	0%	1	20%	
Decline	4	3	19%	1	20%	
**Clinical characteristics**						
**Yr Diagnosed**						
2016	1	1	6%	-	-	
2017	0	0	0%	-	-	
2018	3	3	19%	-	-	
2019	4	4	25%	-	-	
2020	5	5	31%	-	-	
2021	1	1	6%	-	-	
**Distance to PCP**						
<15 Min	9	7	44%	2	40%	
15-30 min	7	5	31%	2	40%	
31-45 min	4	3	19%	1	20%	
No PCP	1	1	6%	0	0%	
**Distance to oncologist**						
<15 min	4	3	19%	1	20%	
15-30 min	12	10	63%	2	40%	
31-45 min	2	1	6%	1	20%	
45min-1hr	1	0	0%	1	20%	
1-2hrs	2	2	13%	0	0%	
**Stage of Cancer**						
0	1	1	6%	-	-	
1	3	3	19%	-	-	
2	2	2	13%	-	-	
3	3	3	19%	-	-	
4	2	2	13%	-	-	
Not Sure	5	5	31%	-	-	

**Table 2:** Subthemes and exemplar quotes corresponding to the frustrations and uncertainty regarding unexpected barriers theme.

Subtheme: Barriers to adhering to clinical posttreatment survivorship care recommendations	
Speaker	Quote
Survivor	“I don’t blame it on the doctor any… but I think they could have put a little effort or give me a list.”
Survivor	"The problem was they were there and I was here. But nowadays, that shouldn't matter…There's telephones. There's all kinds of technology."
Survivor	"You know, I- I’m just afraid, you know, they’re going to turn around and say, 'Hey, uh, we’re going to reschedule your- your, um, biopsy.'"
Survivor	"I had the CT, the follow-up with them, and then he says, '…So I’m pretty much done with you, now I’m going to send you off to somebody else.'…And then I haven’t heard from them since. …"I was kind of like, um, 'Are you kidding me?"'
Caregiver	“[The provider] was two hours late…But we knew we were right because we - we - both - both of us had it written down on our calendars.”
Caregiver	"If there’s a concern I go to the doctor, but the doctor does not understand what the nurse says."
Subtheme: Barriers to daily living	
Survivor	“you don't really realize that you're gonna need that help with your food, and eating, and your bedsheets, and getting in and out of the bath or whatnot, or going on - doing your errands, picking up your medicine.
Survivor	"Like, it's very hard to get in and out of the shower, and, so - uh, I love a hot bath…I can't do that anymore because I can't get in and out"
Survivor	"I can’t golf, I can’t do a lot of things that I used to do, but I can still go out on my deck and read"
Survivor	"[My daughter] is a nurse, and she’d come and make sure that I had my bath and helped me up and make sure I ate… I had a hard time even getting out of bed for a while"
Survivor	"Right now, I'm…in the process of talking to the social worker about maybe getting me…somebody to come in and maybe clean my house …that's where I could really use a little bit of help"
Caregiver	"I think that I mean, I made many meals for her. Um, because, you know, when they're done with their treatments and they go home, they don't feel well. They're not going to cook for themselves. Um, so I think definitely nutrition is important as well because they need that nutrition to build their body back up”
Caregiver	"Um, because, you know, when they're done with their treatments and they go home, they don't feel well. They're not going to cook for themselves."

**Table 3:** Subthemes and exemplar quotes corresponding to the Strategies to improve delivery of posttreatment information theme.

Subtheme: Communication needs to be clear and specific	
Speaker	Quote
Survivor	“He’s very good about sitting there and explaining everything and what will be done, how things will be done…He will sit there and explain, answer questions that I might have.”
Survivor	"There was no, you know, this is what we got to do, or this is, uh, you know, our guidelines. It - it was, uh, very seat-of-the-pants, at the same point in time, very professional"
Survivor	"I understand that you can't predict what's going to happen to everybody. However, I wished that I, you know, had been told, if you're going to have difficulty breathing, then you will be given an inhaler, or you can have respiratory therapy"
Caregiver	"I think they have got a great relationship. And I think, um, he never sugarcoats anything for her, which I think is great. ..Because I think that, you know, he knows she can handle it."
Caregiver	"I just want to know the percentages. And then after we're done, I want to know the outcome, what to expect"
Caregiver	"the things that we had to do, and what we had to take care of, and how we had to do it…they actually explained it really, really well… it was topnotch."
Subtheme: Modes of communication were diverse but needed to be convenient and direct	
Survivor	"It's just when I go to the doctor's office, if there's anything special they want me to do, they tell you…And I follow - I follow what they tell me and they’ll do follow up calls. "
Survivor	"Well, during the pandemic, I guess it was easier for me…To be able to do it over the phone… I didn't want to get out and move around too much."
Survivor	"If I had any questions or anything, um, I can call the girls there. They’re outstanding nurses. And, uh, I feel like they’re right here. …if I need one of them, I call, and I get an answer to what I need. I've never had a problem."
Caregiver	“I would get a hold of [the nurse coordinator]…and tell her about the wound. And.. what was nice, I snapped some pictures of it and I sent it to her. ..She said, yeah, that's - that’s the way it should be.”
Caregiver	"it was in layman's terms, I want to say… keep it simple, stupid, you know, because the simpler, the simplified it is, the easier it [is]"
Caregiver	"They have access to your medical records, …they work out a program for you….So having that ability to kind of all have it intertwined….just having all those resources, you know, um, helped her in her care"

**Table 4:** Subthemes and exemplar quotes corresponding to the strategies to remain positive and respond to emotional concerns of survivorship care theme.

Subtheme: Self-reliance	
Speaker	Quote
Survivor	“If I my family - I mean, like I say, I just don’t want to burden anybody. I’m too strong for it. I don’t sound very strong right now, but I really am.”
Survivor	"I could feel myself getting kind of depressed, and I can't allow me to stay in that state of mind…you have two choices; you can have a good day, or you can have a bad day.”
Survivor	"And I felt like I was making progress pretty much on my own. Um, and again, it's just the way my cancer turned out."
Survivor	"It drives me crazy as somebody comes in and wants to help and they don't do it my way…I can't help it."
Survivor	"I [am] just - not a giver-upper. I just do what I’m told… sometimes it's very hard, but I know what to do."
Caregiver	“Sometimes support groups are not success stories”
Subtheme: A responsive care team	
Survivor	“My whole team…the way they was treating me, it make me… feel that they they work with me.”
Survivor	“Uh, they made us at ease. They made us feel that we weren't alone. And they were top notch.”
Survivor	“And [my provider] wasn't telling me to take these tests just because to make a bunch of money…You know, he was telling me to take these tests because he cared.”
Survivor	“Anyone who walked in, anybody you talked to, anybody that you had question with, they was right there to answer it."
Survivor	"If I cried, they were there to, uh, support me… I would just hope that every other cancer patient has that same treatment."
Survivor	“My, uh, oncologist always hopeful, gives me hope that I'm doing good, and that there's other things that can be done if it does come back.”
Caregiver	"Anytime they talked about it, it was always this, 'Yeah, we can kick its butt. We can do this.'"
Subtheme: Strong social networks	
Survivor	"It kind of gets lonely…what really hurt me was this COVID because it put me in isolation."
Survivor	"I would just go to, uh, close friends, you know. And there are only maybe six… But I won't go with any - a big group because of COVID….I won' go to church."
Survivor	"If I’m eating correctly, and if I'm not, my one sis brings me cabbage rolls. I know I - she’s know I like them. But, uh, they - they just pretty much check in to see if I'm sleeping. …they're concerned, me walking up and down the stairs”
Survivor	"It seems like someone in my family always comes through if I am going through some tough times."
Survivor	" Um, I mean, uh, the support that you’re getting, it's more important. To me, it was, uh, the best medicine, uh, because, uh, my family…, to be - uh, to have the support of my family, and most important, my husband's, that he was there for me all the time, all the way. "
Caregiver	"[Family] support system is very important. …she has a lot of family. She has a good support system. Uh, I think that if - having support be in line with your process."

**Table 5:** Subthemes and exemplar quotes corresponding to the impact of engaging and patient-centered care teams theme.

Subtheme: Pain management	
Speaker	Quote
Survivor	“I kept telling [my surgeon] I was in so much pain, and he didn't want to give me anymore pain medications. That’s why I wound up in the emergency room”
Survivor	"If it wasn't for my daughter-in-law, I would probably starve to death because I was in a lot of pain."
Survivor	"When they didn't see that I was improving… Then I go to the pain specialist. I should have gone to the paint specialist much earlier."
Survivor	"I had no idea what was going on…the pain just got so horrible. And I went to the - my physician, not the pulmonologist because I - I didn't think it was connected…to my problem. A … the one doctor gave me a …block for pain…which helped immensely."
Survivor	"when I explained to my pulmonologist how severe the pain was, then I said, 'I have to have some relief from this pain,' I’m - I couldn't go to the bathroom; I - I couldn't sit; I couldn't stand; I couldn't do anything."
Caregiver	“And it really hurts me to see her suffering like that…And just now we're dealing with pain…The cancer is in remission. And now we're dealing with the pain doctors."
Subtheme: Stress management	
Survivor	“he would tell me that even - not to worry, even if it did come back, there's other things that can be done. And I mean, just made me feel more secure instead of having to worry about it all the time."
Survivor	"So I was still in pain. But I felt under control….You know, if felt like somebody was looking out for me…she said, 'Well, wait a couple of weeks." I said, "No, I want to talk to you every week until I get out of this.'"
Survivor	"The first time…you feel lost. You don't know … what’s going to happen. Like you have the help, uh, you know, from …the medical team that you have. So they, uh, explain… they explained to us, uh, what we're expecting, …step by step"
Survivor	"But every time I go for a scan, it’s like, okay, is this the month that it's gonna say that it's started again?…So now I have to go back in September. So it's like that's sits in my mind all the time."
Survivor	"I don't know what I’m gonna do if it comes back…if I get more treatments, I’m 75 years old."
Caregiver	"When somebody has cancer…. they're thinking, oh my gosh, is this the end of my life….what is my family gonna do? And so when, you know, you get the reassurance from doctors and nurses, you know, and of course, there’s family assurance too, it - it makes it a little bit better."
Caregiver	“They never had a bit of -- what I want to say -- doubt in their - in their words.”
